# Abnormalities of functional brain networks in pathological gambling: a graph-theoretical approach

**DOI:** 10.3389/fnhum.2013.00625

**Published:** 2013-09-27

**Authors:** Melanie Tschernegg, Julia S. Crone, Tina Eigenberger, Philipp Schwartenbeck, Mira Fauth-Bühler, Tagrid Lemènager, Karl Mann, Natasha Thon, Friedrich M. Wurst, Martin Kronbichler

**Affiliations:** ^1^Centre for Neurocognitive Research and Department of Psychology, University of SalzburgSalzburg, Austria; ^2^Neuroscience Institute and Centre for Neurocognitive Research, Christian-Doppler-Klinik, Paracelsus Medical University SalzburgSalzburg, Austria; ^3^Department of Psychiatry and Psychotherapy II, Christian-Doppler-Klinik, Paracelsus Medical University SalzburgSalzburg, Austria; ^4^Department of Addictive Behavior and Addiction Medicine, Central Institute of Mental Health, University of HeidelbergMannheim, Germany

**Keywords:** fMRI, graph theory, network, connectivity, pathological gambling, reward, behavioral addiction, small world

## Abstract

Functional neuroimaging studies of pathological gambling (PG) demonstrate alterations in frontal and subcortical regions of the mesolimbic reward system. However, most investigations were performed using tasks involving reward processing or executive functions. Little is known about brain network abnormalities during task-free resting state in PG. In the present study, graph-theoretical methods were used to investigate network properties of resting state functional magnetic resonance imaging data in PG. We compared 19 patients with PG to 19 healthy controls (HCs) using the Graph Analysis Toolbox (GAT). None of the examined global metrics differed between groups. At the nodal level, pathological gambler showed a reduced clustering coefficient in the left paracingulate cortex and the left juxtapositional lobe (supplementary motor area, SMA), reduced local efficiency in the left SMA, as well as an increased node betweenness for the left and right paracingulate cortex and the left SMA. At an uncorrected threshold level, the node betweenness in the left inferior frontal gyrus was decreased and increased in the caudate. Additionally, increased functional connectivity between fronto-striatal regions and within frontal regions has also been found for the gambling patients. These findings suggest that regions associated with the reward system demonstrate reduced segregation but enhanced integration while regions associated with executive functions demonstrate reduced integration. The present study makes evident that PG is also associated with abnormalities in the topological network structure of the brain during rest. Since alterations in PG cannot be explained by direct effects of abused substances on the brain, these findings will be of relevance for understanding functional connectivity in other addictive disorders.

## INTRODUCTION

Patients suffering from pathological gambling (PG) show persistent gambling behavior despite negative consequences resulting in a wide-range of psychosocial impairments (Goudriaan et al., 2004). PG is classified as an impulse control disorder in DSM-IV (American Psychiatric Association, 2000), but is increasingly conceptualized as a behavioral addiction with striking similarities to substance addictions such as withdrawal symptoms and signs of tolerance (Petry, 2007). Therefore, PG (besides being renamed as disordered gambling) has been reclassified under the chapter “Addiction and related disorders” (together with substance addictions) in DSM 5 (American Psychiatric Association, 2013; Petry et al., 2013).

Most functional neuroimaging studies in PG up to date have examined brain activity abnormalities using paradigms such as reward processing, reactivity to gambling related cues, learning, decision making, and executive functions (for reviews, see Potenza, 2008, 2013; van Holst et al., 2010). In line with brain imaging studies on substance addiction, activation abnormalities in regions of the mesolimbic reward system (mainly in orbitofrontal, medial and lateral prefrontal regions, and the ventral striatum) were consistently found in patients with PG (Cavedini et al., 2002; Potenza et al., 2003; Reuter et al., 2005; Tanabe et al., 2007; Balodis et al., 2012; Choi et al., 2012; Miedl et al., 2012; van Holst et al., 2012a; Hudgens-Haney et al., 2013; Limbrick-Oldfield et al., 2013).

Brain activation differences in fronto-striatal regions in PG have also been found in executive function tasks and been commonly interpreted as reflecting impairments in cognitive control and inhibitory functions (Potenza et al., 2003) which contribute to maladaptive decision making in PG, comparable to such impairments in substance addiction (Tanabe et al., 2007).

Recent interest in functional neuroimaging studies on neuropsychiatric disorders has focused on analyzing resting state functional connectivity (Fox and Greicius, 2010; van den Heuvel and Hulshoff Pol, 2010; Menon, 2011; Xia and He, 2011; Buckholtz and Meyer-Lindenberg, 2012; Yu et al., 2012). Compared to task based studies, resting state data is easier to obtain and does not have to deal with group differences in task performance and compliance. Resting state connectivity studies have revealed abnormalities in a wide range of neuropsychiatric disorders such as depression, schizophrenia, attention-deficit hyperactivity disorder (ADHD), and Alzheimer’s disease (for review, see Greicius, 2008).

Resting state functional magnetic resonance imaging (fMRI) data can also be used to analyze topological network properties of the brain using graph-theoretical approaches (He and Evans, 2010; Bullmore and Sporns, 2012). These approaches provide important information on the architecture of brain networks. Small-world networks are characterized by dense local interconnectivity and short path length linking individual network nodes in a short and efficient way (e.g., brain regions based on a parcellation atlas; Bullmore and Sporns, 2009). Short pathways between one node and any other node as well as a high density of connections between nearest neighbors are necessary for efficient segregation and functional integration (Salvador et al., 2005; Achard et al., 2006; Bassett and Bullmore, 2009). Network graphs are based on structural or functional data and quantify the structural and functional organization of the brain (Stam and Reijneveld, 2007).

Studies have shown that the small-world architecture and topological network properties of the brain exhibit abnormalities in neuropsychiatric disorders (e.g., He et al., 2009; Lynall et al., 2010; Zhang et al., 2011a; Cocchi et al., 2012; Cisler et al., 2013; for a review, see Xia and He, 2011). For example, patient with schizophrenia show lower cortical integration (lower amount of connections, longer path lengths, and lower clustering coefficients) in the frontal, parietal, and temporal pole (Liu et al., 2009). Zhang et al. (2011a) found global integration differences between HCs and patients with major depressive disorder and differences in nodal centrality for frontal areas, and regions of the default-mode network as well as for subcortical regions like the caudate. Furthermore, patients with obsessive-compulsive disorder (OCD) show altered functional connectivity and small worldness-properties (Zhang et al., 2011b). OCD patients demonstrate higher local clustering in the brain’s cognitive control network (posterior temporal regions and the cingulate cortex). Differences in brain topology are also reported for young adults with ADHD (Cocchi et al., 2012). Functional segregation of the orbitofrontal cortex in the intrinsic brain network is enhanced in ADHD which can be linked to attentional and perceptual control deficits. Both approaches demonstrate how network analyses of the brain identify alternations directly related to symptoms of the specific mental disorder.

To date, comparatively less is known about resting state functional connectivity in addictive disorders (for review, see Sutherland et al., 2012). For example, a resting state fMRI study in chronic heroin addicts found increased functional connectivity of mesolimbic pathways and decreased functional connectivity between frontal areas (Ma et al., 2010). Two studies using graph-theoretical approaches reported differences in global small-world properties and an increased degree in a number of medial frontal, frontal, and subcortical regions in chronic abstinent heroin addicts (Liu et al., 2009; Yuan et al., 2010). These studies suggest that topological network properties may provide important insights in functional brain abnormalities in addiction. However, both of these studies on small-world properties in addiction had a relatively small sample size (11 patients in Liu et al., 2009 and 12 patients in Yuan et al., 2010). Furthermore, in studies investigating substance addiction, results may also partly reflect the effects of the abused substance on brain structure and function (Clark and Limbrick-Oldfield, 2013).

To our knowledge, not one single study on resting brain connectivity and especially on topological network properties has been conducted in PG. Two recent reports of white matter microstructural abnormalities in PG suggest that brain connectivity and network organization may be affected in PG (Joutsa et al., 2011; Yip et al., 2013). Two studies in internet addiction report functional connectivity abnormalities (Ding et al., 2013; Hong et al., 2013). Ding et al. (2013) report differences between controls and internet addicts in functional connectivity between a part of the default mode network, that is, the posterior cingulate cortex (PCC), and regions in the cerebellum, the inferior parietal lobule, and the middle temporal gyrus. Hong et al. (2013) report decreased connectivity in internet addiction between a number of cortical and subcortical regions but no significant group differences in topological network properties. The authors point out that the low number of participants (11 addicted adolescents and 11 matched HCs) could be a reason for the absence of statistically significant differences in network properties.

The aim of the present study is to provide first evidence for alterations in topological network properties using resting state fMRI and gain further insights on the neural correlates of this disorder and addictive disorders in general.

## MATERIALS AND METHODS

### SUBJECTS

This study has been approved by the local ethics committee. Nineteen patients with PG and 19 age-matched HCs with no history of neurological or psychiatric disorders participated in this study. Written informed consent was provided by all participants. All patients were seeking treatment and have been recruited at the Pathological Gambling out-patient clinic at the Department of Psychiatry and Psychotherapy II. Control subjects were recruited via advertisements and mailings.

### BEHAVIORAL ASSESSMENT

The German version of the short questionnaire on gambling behavior (Kurzfragebogen zum Glücksspielverhalten – KFG; Petry, 1996) and The South Oaks Gambling Screen (SOGS) by Lesieur and Blume (1987) were used to quantify gambling behavior. Furthermore, all participants completed the Alcohol Use Disorders Identification Test (AUDIT; Babor et al., 2006), the Fagerstrom Test for Nicotine Dependence (FTND; Fagerstrom, 1978), the Behavioral Inhibition Scale (BIS; Carver and White, 1994), and the Beck Depression Inventory (BDI; Beck et al., 1996).

### fMRI DATA ACQUISITION PREPROCESSING

Resting state fMRI was performed with a 3 Tesla Siemens Tim Trio MRI using a 32-channel head coil. All participants were asked to quietly rest in the scanner with their eyes closed and not to think of anything specific. Two-hundred and fifty T2*-weighted images were acquired (including six dummy scans which were discarded) with a gradient echo-planar imaging sequences with the following parameters: TR: 2.25 s; TE: 30 ms; flip angle: 78°; field of view (FOV): 192 mm × 192 mm; matrix size: 64 × 64; 36 slices; slice thickness: 3 mm; slice gap 0.3 mm; voxel size: 3 mm × 3 mm × 3 mm. Additionally, a high-resolution structural scan (sagittal T1-weighted MPRAGE sequence; TR: 2300 ms; TE: 2.91 ms; voxel size: 1 mm × 1 mm × 1.2 mm; slice thickness: 1.20 mm; FOV: 356 mm × 356 mm; 160 slices; flip angle: 9°) and fieldmaps were obtained from each participant.

Functional magnetic resonance imaging data were preprocessed using Statistical Parametrical Mapping (SPM 8, Wellcome Department of Imaging Neuroscience, London, UK^1^). The following procedures were included: realignment and unwarping to compensate for movement-related artifacts; slice timing correction; co-registration of the EPI scans to the skull-stripped T1-weighted structural scan; normalization to standard stereotaxic anatomical Montreal Neurological Institute (MNI) space; smoothing with 6 mm full-width at half-maximum (FWHM) Gaussian kernel; voxel size was resampled to isotropic 3 mm × 3 mm × 3 mm.

To address the problem of confounds due to small head motion which may influence resting state connectivity, we ensured that all data sets did not exhibit movements larger than 3 mm for translations or 3° for rotations. Movement parameters were compared between patients and HCs using two-tailed *t*-tests. There are no significant differences in any of the six movement parameters (all *t*s < 1, all *p*s > 0.3).

For further analyses, noise correction and filtering with a bandpass filter between 0.01 and 0.1 Hz was performed with the conn toolbox (Whitfield-Gabrieli and Nieto-Castanon, 2012). For noise correction all six movement parameters and the first derivative of the time-series were removed from the data by regression. For further noise reduction, noise signals were estimated from white matter and CSF signal and removed from the data with the CompCor method (Behzadi et al., 2007) as implemented in the conn toolbox. These noise removal steps have been shown to substantially reduce noise from non-neural sources and increase the sensitivity and reliability of functional connectivity analysis (Whitfield-Gabrieli and Nieto-Castanon, 2012). No global signal regression was performed as it may result in lower reproducibility of network metrics (see Telesford et al., 2013).

### NETWORK CONSTRUCTION

The Harvard–Oxford Atlas was used to extract the preprocessed fMRI data from 48 left and 48 right hemisphere cortical regions, as well as from seven left and seven right subcortical regions. Time-series of the low-frequency BOLD signal were extracted for each of the 110 regions and averaged over all voxels in each node. For each subject, the time-series of all 110 regions were correlated with each other to create an undirected and weighted correlation matrix using Pearson correlation. These steps were performed with the conn toolbox. In contrast to partial correlation, the Pearson correlation coefficient is gaining higher values of reproducibility (see Telesford et al., 2013). In this network, each region represents a node with the correlation coefficients of the time-series between the different regions defining the edges resulting in a 110 × 110 connectivity matrix.

### GRAPH ANALYSES

Analyses of network properties were performed with the GAT^2^ (Hosseini et al., 2012), which uses routines of the Brain Connectivity Toolbox for network metrics calculation (Rubinov and Sporns, 2010).

#### Threshold selection

To make groups comparable, we ensured that all graphs had the same number of edges by applying an individual threshold to each correlation matrix. This was done by calculating the ratio of the number of actual connections divided by the maximum number of all possible connections described as the so-called cost of the network (connection density). Since there is still no consensus of the best threshold to be chosen, a wide range of threshold values were applied in this study (0.11 ≤ *T* ≤ 0.55 with an increment of 0.02). To verify that the selection of the threshold range is not too wide which may produce disconnected nodes and networks without small worldness features on either ends of the range, we ensured that all subjects (a) had an averaged degree value of 2*log(*N*) with *N* = number of nodes and (b) showed network properties of small worldness with σ > 1.1 in all threshold values (Zhang et al., 2011a).

#### Network metrics

For each threshold, the following global metrics were calculated: characteristic path length (*L*); the average of the clustering coefficient (*C*); global efficiency (*E*_glob_); small worldness (σ); additionally, the following local metrics were calculated for each threshold: degree (*k*); local efficiency (*E*_loc_); node betweenness (*N*_bc_); clustering coefficient (*C*).

The degree describes the number of edges linking one node to the rest of the network and gives information on how functionally connected a network is. The clustering coefficient is a measure of degree to which nodes in a graph are forming a cluster. The characteristic path length describes the number of edges between one node and any other node in a network giving an overview of the effectiveness of information transfer. The global efficiency is inversely related to the characteristic path length. The local efficiency is computed on node neighborhoods and is related to the clustering coefficient reflecting the efficiency of parallel information transfer, robustness, and fault tolerance of a network. Compared to the clustering coefficient and the characteristic path length, measures of efficiency have the advantage of including disconnected nodes with a value of 0 while the former remove them from the analysis, and therefore, may falsify the results when disconnected nodes are present (Achard and Bullmore, 2007). The node betweenness is a measure of centrality and specifies the fraction of all shortest pathways in a network that contain a given node. The so-called small worldness is the ratio of the averaged and normalized clustering coefficient (γ) to the normalized characteristic path length (λ) and assesses the small-world properties of a network characterized by high clustering coefficient and a low characteristic path length. Small-worldness properties of a network are usually given when sigma (σ) is greater than 1.

All metrics were compared with the corresponding values obtained and averaged from 20 random networks with the same number of nodes, total edges, and degree distribution resulting in, for example, γ = *C/C*_rand_ and λ = *L/L*_rand_ (Maslov and Sneppen, 2002).

#### Statistical analyses

Group comparisons of the metrics were conducted with permutation tests implemented in the GAT toolbox using the area under the curve (AUC) calculated over the threshold for each metric (Bruno et al., 2012; Hosseini et al., 2012; Singh et al., 2013). All results were corrected for multiple comparisons using a false positive correction, *p* < 1/*N* (Alexander-Bloch et al., 2010). All *p*-values corrected for multiple comparisons have been transformed and are reported as *p*_cor_.

Since this study is an exploratory study and the first in PG using graph theoretical approaches to assess network properties in resting state data, we also report significant results with uncorrected *p*-values.

To examine possible alterations of functional connectivity strength between regions, the correlation values of all regions were compared between both groups to find significant differences in connectivity. Analyses of functional connectivity were performed with the conn toolbox and corrected for multiple comparisons using an FDR-threshold, *p* < 0.05.

## RESULTS

### SAMPLE CHARACTERISTICS

Sample characteristics are shown in **Table 1**. No statistically significant group differences were found for sex ratio, years of education, or age. Furthermore, PG patients were comparable to HCs with respect to tobacco and alcohol consumption as assessed by the FTND and the AUDIT.

**Table 1:** Sample characteristics and group differences for healthy controls (HCs) and pathological gamblers (PG) in all questionnaires.

	HC		PG	
*N*/female	19/2		19/2	
*N* smoker	4		6	
	**Mean**	**SD**	**Mean**	**SD**
Age	42.4	14.85	41.4	10.35
AUDIT	3.63	2.53	5.07	6.06
BDI	3.53	5.20	13.72	12.9^***^
KFG	2.22	5.52	36.94	9.49^***^
SOGS	0.54	1.45	10.79	2.69^***^
BIS	48.93	12.91	67.48	9.59^***^

*** *p* < 0.001.

Large group differences were found in gambling behavior (KFG; SOGS). PG patients also demonstrated a larger number of depressive symptoms as measured by the BDI and higher impulsivity as measured by the BIS.

### GLOBAL METRICS

**FIGURE 1:**
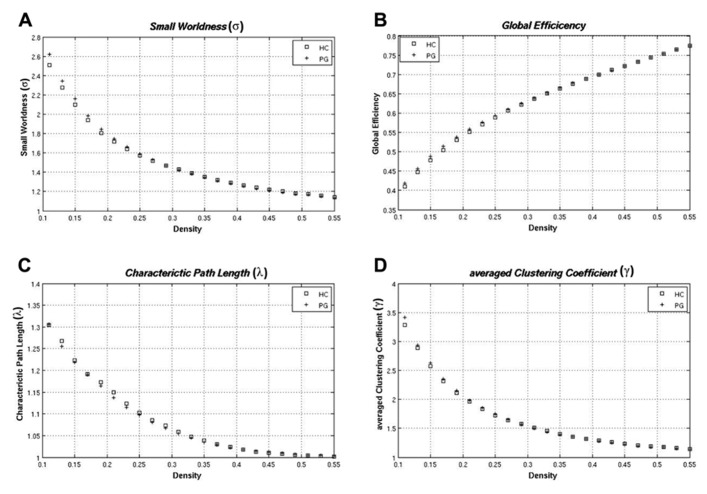
**Global metrics for pathological gamblers (PG) and healthy controls (HC) in all density thresholds: (A)** small worldness (σ); **(B)** global efficiency; **(C)** characteristic path length (λ); **(D)** averaged clustering coefficient (γ).

Both groups showed small worldness properties with σ > 1 and there were no significant differences between groups (*p* = 0.845). Compared to random networks, both groups showed a higher averaged clustering coefficient (γ > 1) and similar values for the characteristic path length (λ ~ 1). None of the global metrics differed between patients and controls (*E*_glob_: *p* = 0.646; λ: *p* = 0.797; γ: *p* = 0.817). Results for all global metrics are displayed in **Figure 1**.

### NODAL METRICS

**FIGURE 2:**
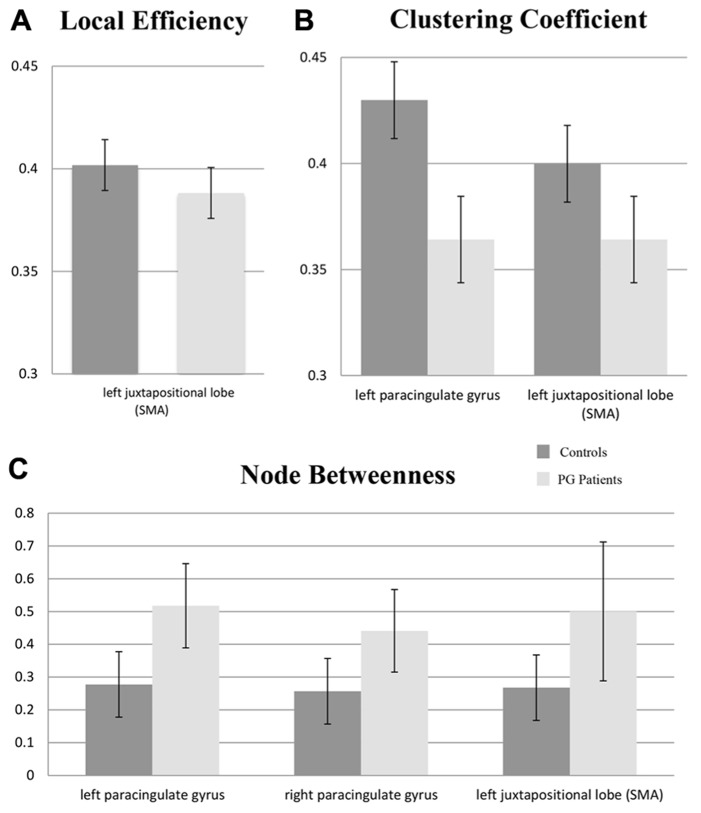
**Mean area under the curve (AUC) values for nodal metrics in regions with statistically significant group differences (*p*_cor_ < 0.05) between controls and patients with pathological gambling (PG): (A) local efficiency; (B) clustering coefficient; (C) node betweenness.** Error bars reflect standard deviations; SMA: supplementary motor area.

At the corrected significance threshold, differences in nodal metrics were found in medial frontal regions. As can be seen in **Figure 2**, patients with PG demonstrated a decreased clustering coefficient for the left juxtapositional lobe (supplementary motor area, SMA; *p*_cor_ = 0.038) and the left paracingulate gyrus (*p*_cor_ = 0.044). Additionally, local efficiency for the left juxtapositional lobe (SMA) was decreased for PG patients (*p*_cor_**= 0.022). Node betweenness was increased in the right paracingulate gyrus (*p*_cor_ = 0.05) as well as in the left paracingulate gyrus (*p*_cor_ = 0.011) in PG patients. Further differences in regional metrics at an uncorrected significance level are shown for exploratory purposes in **Table 2**.

**Table 2:** Significant differences in all metrics using area under the curve (AUC) for pathological gamblers (PG) and healthy controls (HCs).

Hemisphere	Region	Metric	*p*-Value	*p*_cor_	Group comparison
Right	Paracingulate	NB	0.009	0.050^*^	PG > HC
	Insular	LE	0.057	0.316	HC > PG
		NB	0.027	0.150	PG > HC
	Precentral	CC	0.026	0.144	HC > PG
	Supramarginal (anterior)	NB	0.019	0.105	HC > PG
	Temporal fusiform (posterior)	DG	0.022	0.122	PG > HC
	Temporal fusiform (anterior)	LE	0.031	0.172	PG > HC
		NB	0.040	0.222	HC > PG
	Caudate	NB	0.041	0.227	PG > HC
	Hippocampus	NB	0.036	0.200	HC > PG
Left	Juxtapositional (SMA)	CC	0.007	0.038^*^	HC > PG
		LE	0.004	0.022^*^	HC > PG
		NB	0.041	0.227	PG > HC
	Paracingulate	CC	0.008	0.044^*^	HC > PG
		LE	0.033	0.183	HC > PG
		NB	0.002	0.011^*^	PG > HC
	Inferior frontal (pars triangularis)	NB	0.026	0.144	HC > PG
	Middle temporal (anterior)	CC	0.039	0.216	PG > HC
		DG	0.013	0.072	HC > PG
	Middle temporal (temporoocci)	DG	0.024	0.133	PG > HC
		NB	0.037	0.205	PG > HC
	Inferior temporal (anterior)	CC	0.034	0.188	PG > HC
		LE	0.035	0.194	PG > HC
	Inferior temporal (temporoocci)	NB	0.034	0.188	PG > HC
	Lateral occipital superior	CC	0.025	0.138	HC > PG
		LE	0.016	0.088	HC > PG
	Temporal fusiform (posterior)	CC	0.045	0.250	PG > HC
		LE	0.024	0.133	PG > HC

*CC, clustering coefficient; DG, degree; LE, local efficiency; NB, node betweenness; *p*_cor_: corrected for multiple comparisons;*

* Statistically significant at *p* < 0.05, corrected for multiple comparisons.

### FUNCTIONAL CONNECTIVITY ANALYSES

Functional connectivity was increased in patients between frontal regions and between frontal and temporal regions (see **Table 3**). Furthermore, we found increased connectivity in patients between the left caudate and the right anterior cingulum as well as the left anterior cingulum. Additionally, the left amygdala with the left subcallosal cortex demonstrated weaker connectivity in patients than in controls.

**Table 3:** Significant differences between pathological gamblers (PG) and healthy controls (HC) in functional connectivity.

Region	Region	Group	*p*-Value^*^
Right inferior frontal (pas opercularis)	Right hippocampus	HC < PG	<0.001
Right frontal operculum	Right inferior temporal gyrus (temporoocci)	HC< PG	<0.001
Right frontal operculum	Right cuneal cortex	HC < PG	<0.001
Right frontal operculum	Right temporal fusiform (anterior)	HC < PG	0.003
Right frontal operculum	Left inferior temporal (temporoocci part)	HC < PG	0.001
Right frontal operculum	Left middle frontal	HC < PG	<0.001
Right frontal operculum	Left middle temporal (temporoocci)	HC < PG	<0.001
Right frontal operculum	Left frontal pole	HC < PG	0.003
Left caudate	Right cingulate (anterior)	HC < PG	0.001
Left caudate	Left cingulate (anterior)	HC < PG	0.004
Right angular	Left lateral occipital (superior)	HC < PG	<0.001
Right temporal fusiform (posterior)	Left inferior temporal (temporoocci)	HC < PG	0.001
Left middle temporal (anterior)	Left parahippocampal (anterior)	PG < HC	<0.001
Left subcallosal	Left amygdala	PG < HC	0.004

* Statistically significant at FDR-corrected threshold, *p* < 0.05.

## DISCUSSION

In this exploratory study, we investigated the functional network properties of patients with PG during the resting state using a graph-theoretical approach. While several studies could demonstrate functional abnormalities in PG during tasks associated with gambling, executive functions, and reward processing (Reuter et al., 2005; Tanabe et al., 2007; Balodis et al., 2012; Choi et al., 2012; Miedl et al., 2012; van Holst et al., 2012a; Hudgens-Haney et al., 2013; Limbrick-Oldfield et al., 2013; for a review, see Potenza, 2013), we are the first to show that patients with a behavioral addiction such as PG exhibit alterations in the topology of resting state networks in regions associated with reward processing and self-regulation.

Network properties at the global level showed no differences between patients and HCs. Global efficiency of information transfer and fault tolerance, for example, were similarly high in both groups. This is in line with a previous graph-theoretical study investigating the global topology of subjects suffering from internet addiction (Hong et al., 2013).

In contrast to global network properties, we found significant differences between healthy subjects and patients in network properties at the nodal level. Corrected for multiple comparisons, only medial frontal regions were affected in patients with PG. The SMA and the paracingulate cortex both showed a reduced clustering coefficient and impaired local efficiency of information transfer and fault tolerance. Furthermore, the contribution to the number of shortest paths was increased in both regions suggesting that these regions seem to adopt a more central position in the network than in healthy subjects. Note that the results for local efficiency in the paracingulate cortex and for betweenness centrality in the SMA are only tendencies, since they are not significant at a corrected level. These findings indicate that in medial frontal regions the balance between integration and segregation seem to be altered.

Medial frontal regions like the paracingulate cortex are associated with reward processing (Knutson et al., 2001; van den Bos et al., 2007; Fujiwara et al., 2009). Dysfunctions in reward processing are typical findings of previous investigations in PG (Reuter et al., 2005; Clark and Limbrick-Oldfield, 2013). The cingulate cortex is also important for gambling situations especially for specific processes of gambling (Campbell-Meiklejohn et al., 2008) like loss-chasing and quitting gambling.

Another frontal region which was found to be affected in PG is the SMA. The SMA demonstrated the same pattern of impairments as the paracingulate cortex with decreased clustering and efficiency of local information transfer but an increase in betweenness centrality.

The SMA is associated with motor execution and vigilance performance (Hinds et al., 2013) but is also involved in error detection and reward expectancy (McClure et al., 2004). Thus, the findings of this study demonstrating alterations in integration and segregation of medial frontal regions may underlie specific behavioral difficulties patients with PG exhibit.

Since this was an exploratory study, we also want to discuss findings which do not exceed the threshold selected to correct for multiple comparisons.

We found a reduced fraction of path length in the left inferior frontal gyrus which also contributes to the general findings of impairments in frontal regions in gambling and addiction. A previous study showed that PG patients exhibit alterations in inferior frontal activity during gambling cue presentation (Crockford et al., 2005). The inferior frontal gyrus has been associated with executive control and response inhibition (Hampshire et al., 2010). Interestingly, while medial frontal regions showed an increase in betweenness centrality, in lateral frontal regions, this metric was decreased. This pattern may support previous findings demonstrating deficits in self-regulation and working memory in PG (Forbush et al., 2008), but enhanced involvement of the reward system.

Additionally, we further found alterations in subcortical regions at an uncorrected threshold level. The right caudate plays a more central role as a main hub for integration of information compared to HCs while the hippocampus is less involved. Again, this points out the enhanced involvement of the reward system in PG. The caudate is part of the striatum which is an important part of the mesolimbic reward system. The alterations found in network properties of the hippocampus, are in line with deficits in heroin addicts identified in a previous study (Liu et al., 2009).

This pattern of impaired topology in regions which were previously associated with the executive control network and the reward system (Potenza et al., 2003; Reuter et al., 2005; Tanabe et al., 2007; Limbrick-Oldfield et al., 2013) is complemented by our findings of increased functional connectivity of fronto-striatal circuits and between frontal regions. Note that almost all differences in connectivity in which patients with PG exhibit higher functional connectivity than controls affect regions associated with the reward system. This is in line with previous studies finding alterations in functional connectivity between medial frontal and subcortical regions in addiction (Ma et al., 2010).

One previous study investigating network properties in behavioral addiction was performed in subjects with internet addiction (Hong et al., 2013). This study did not identify any alterations in the network topology in addicts. However, the authors emphasize that the non-significant results may be due to the small sample size. Additionally, two studies in heroin addiction also focused on graph-theoretical methods to investigate network properties (Liu et al., 2009; Yuan et al., 2010). They report dysfunction in several frontal regions including the cingulate cortex and the SMA, and subcortical regions including the striatum and the hippocampus. Our findings endorse the association of addictive behavior with alterations in functional connectivity and network topology during resting state in these specific frontal and striatal regions. This finding is of high relevance since previous investigations showing abnormalities in brain topology focused on addiction involving substance abuse. Thus, conclusions drawn from these studies are confounded by the neurotoxic effects of the abused substances (Clark and Limbrick-Oldfield, 2013). With this study, we confirm that abnormalities in network properties can also be found in behavioral addiction and therefore cannot solely be explained by effects of drugs on brain connectivity.

There are no available standards for a uniform application of graph theories at present (Bullmore and Sporns, 2009). One methodological limitation when investigating network topology with a graph-theoretical approach, for example, is the choice of thresholds. There are several possibilities to select the threshold and no golden standard has been defined yet. When comparing groups it should be ensured that each network has the same number of edges. However, the problem with a global threshold is that it may lead to disconnected graphs. Comparing network properties of one graph with the other is problematic if the one is connected at a given node and the other is disconnected. To address this problem, we ensured that the averaged degree is above the selected threshold and all subjects show small-world properties. Furthermore, we also investigated the global and local efficiency in addition to the clustering coefficient and the characteristic path length. These metrics have some methodological advantages when dealing with disconnectedness (Achard and Bullmore, 2007).

Another limitation is the wide range of thresholds selected. Depending on the range, results differ between studies and make comparison of findings and their interpretation difficult. However, we have implemented strategies which have been successfully applied in previous studies using graph-theoretical approaches (e.g., Zhang et al., 2011a; Bruno et al., 2012). Since this is a first exploratory study in PG using graph-theoretical analyses of resting state fMRI data, further research must be conducted to confirm these results.

Moreover, Zalesky et al. (2012) has shown that the type of randomization (topology randomization, correlation matrix randomization, or time-series randomization) influences the normalization process of the metrics. For a low density of around 7% the authors identified a discrepancy of approximately 60% when applying topology randomization compared to correlation matrix randomization to estimate the normalized clustering coefficient. In addition, using correlation matrix randomization to normalize characteristic path length may lead to longer path lengths due to the randomization of hub nodes since the degree distribution is not preserved. These limitations affect especially low density thresholds around 7% and are evident when looking at absolute small-world properties of the networks in each group. However, they are less essential for the comparison of network metrics between groups which is the focus of this study.

This first study in PG using graph-theoretical approaches to investigate network properties demonstrates that alterations of regions associated with the reward system and executive functions are not only present in task-related activity but also during rest. Alterations are reflected in a decrease in segregation and an increase of information integration in specific regions of the reward system.

This may contribute to the ongoing discussion whether PG is characterized by a hyper- or a hypoactive reward system (Hommer et al., 2011; van Holst et al., 2012b). Furthermore, our results suggest deficits of integration in regions associated with executive functions. These alterations may provide further explanation for several symptoms and previous findings in PG (for a review see Goudriaan et al., 2004; van Holst et al., 2010)

## Conflict of Interest Statement

The authors declare that the research was conducted in the absence of any commercial or financial relationships that could be construed as a potential conflict of interest.

## Footnotes

1

2
